# Performance of diabetes screening tests: an evaluation study of Iranian diabetes screening program

**DOI:** 10.1186/s13098-021-00632-9

**Published:** 2021-01-26

**Authors:** Fateme Kianpour, Mohammad Fararouei, Jafar Hassanzadeh, Mohammadnabi Mohammadi, Mostafa Dianatinasab

**Affiliations:** 1 Student Research Center, Department of Epidemiology, Shiraz University of Medical Sciences, 7134767617 Shiraz, Iran; 2 Department of Epidemiology, Shiraz University of Medical Sciences, 7134767617 Shiraz, Iran; 3 Health Deputy, Gerash University of Medical Sciences, 7134767617 Gerash, Iran; 4 Department of Complex Genetics and Epidemiology, School of Nutrition and Translational Research in Metabolism, Maastricht University, 6200 MD Maastricht, The Netherlands

**Keywords:** Diabetes mellitus, Screening, HbA1c, Fasting plasma glucose

## Abstract

**Background:**

Type 2 diabetes is a common non-communicable disease that is responsible for about 9% of all deaths and a 25% reduction in life expectancy. However, nearly half of the diabetic patients are not aware of their disease. In this regard, to identify un-known diabetic patients, diabetes screening is of great importance. This study was conducted to evaluate the performance of two commonly used diabetes screening tests that are currently recommended by the Iranian diabetes screening program for (DSP).

**Methods:**

The validity of the two diabetes screening tests were measured among 1057 participants who were older than 30 years of age. The studied screening tests included capillary fasting blood glucose (CBG) and glycated hemoglobin (HbA1c). The golden standard for measuring the validity of the tests was venous fasting plasma glucose (VPG).

**Results:**

According to the results, the sensitivity of CBG and HbA1c tests was 69.01% and 84.5%, and the specificity of the tests were 95.7% and 79.3%, respectively. Positive and negative predictive values were 53.84% and 97.72% for CBG and 22.72% and 98.61% for HbA1c, respectively. The recommended cut points for CBG and HbA1c were 116.5 mg/dl and 7.15%, respectively. Using these values as the new cut points, sensitivity and specificity of CBG and HbA1c changed to 80.30% and 89.10%, and 77.50% and 94.20%, respectively.

**Conclusions:**

Compared to several other countries, the performance of Iranian DSP is relatively better. The Receiver Operating Characteristic Curve suggested new cut points for significantly better performance of DSP.

## Background

Several factors including socio-economic development and significant progress in health and medical cares reduced mortality at a younger age and raised life expectancy of mankind. On the other hand, these changes along with the new sedentary lifestyles caused sharp rises in several chronic diseases [1]. Type 2 diabetes mellitus is a common metabolic disease [2] that about one-half of the patients are unaware of their condition [3]. In addition, about 9% of total deaths and 25% reduction in life expectancy are directly or indirectly associated with diabetes [4, 5]. For example, cardiovascular diseases are among the most common diabetes-related causes of deaths and about 43% of deaths due to diabetes occur among individuals under 70 years of age [6]. This means that type 2 diabetes kills patients when they are still socio-economically active [7]. It is also estimated that about 12% of the global health budget is being spent on diabetes and its related conditions [8]. Apart from the above facts, figures suggest that type 2 diabetes is alarmingly increasing, and is becoming a serious problem threatening global health and economy [9, 10]. Reports suggested that the global prevalence of type 2 diabetes among individuals over 18 years of age was about 9% in 2014 [11]. However, the international diabetes federation (IDF) has estimated that the prevalence of diabetes is yet to raise to 9.9% in 2030 [12]. With regard to the geographical distribution of diabetes, it is estimated that 20% of the world’s diabetic patients are living in South East Asia, and it is predicted that in the near future, the Asian population will be more seriously affected by type 2 diabetes compared to the population of the other parts of the world [13, 14].

As the epidemic of type 2 diabetes is expanding, the costs of the disease, including the cost of diagnosis and treatment of its complications, is also rising sharply. This is because, on average, the cost of treating or controlling the complications among diabetes patients is about 3.2 times higher than the cost of treating non-diabetic patients for the similar conditions [15]. In addition to the high morbidity and mortality due to a wide range of health conditions that are related to diabetes, the economic burden of the disease in different countries is remarkable [16]. As a result, the economic burden of diabetes in low or middle-income countries adversely affects the development of these countries [17]. Despite the very high impact of type 2 diabetes on the health and economy of the world’s population [18], a large number of people with type 2 diabetes are not aware of their condition and, therefore, are exposed to the consequences of the late diagnosis (4 to 7 years) [19, 20] and the serious consequences of the disease. This is due to the nature of the disease, which starts with almost no significant sign and symptom. As a result, screening of high-risk populations is the only feasible way of detecting patients at an earlier stage of the disease. This prevents the appearance of serious and life threatening complications including neuropathy, nephropathy, retinopathy, cardiovascular diseases, and stork [21].

Several methods and strategies are used to conduct diabetes screening programs, of which all are conducted on high risk groups (e.g., adults older than certain ages or those with some defined risk factors such as overweight and positive family history) to make the programs more efficient and affordable. Among different screening and diagnostic tests, capillary fasting blood glucose (CBG), Venous plasma glucose (VPG), and HbA1C [22] are vastly used for population-based screening for diabetes and prediabetes. Nevertheless, there are many validity and reliability considerations about these methods as all have limited sensitivity and specificity and predictive values [18]. For example, a study on Thai population revealed significant controversies over the results of different tests [23]. In addition, although VPG is used as a gold standard for diagnosis of diabetes by several researchers, the necessity of using sophisticated instruments and laboratory settings makes the test less applicable as a screening tool in developing countries [24]. As a result, countries use different criteria and methods for screening and diagnosis of diabetes.

### Diabetes in Iran

Similar to the rest of the world including the Middle East, over the past three decades, the prevalence of diabetes in Iran has been doubled and based on an estimate in 2016, the prevalence of type 2 diabetes in Iran was 10.3% [25]. Reports also suggest that in Iran a huge amount of money is being spent on the treatment of diabetes and its related health problems [8]. In this regard, early detection of type 2 diabetes is essential in the prevention and management of its severe and irreversible complications in Iran [26].

This study is conducted to evaluate the performance of Iranian DSP and its recommended cut points for the selected diabetes screening tests. In particular, this study aimed to evaluate sensitivity, specificity, and predictive values of CBG and HbA1c as the screening tests, using VPG as the gold standard [27].

## Material and methods

### The settings

This study was conducted among rural residences of Gerash county. Located in the southern part of Fars province, Iran, the county consists of 25 villages with about 14,456 rural residents. In the study area, seven health houses and two rural health centers deliver primary health services to the defined population. The participants were assured that their information would be used for research proposes only. Because of the illiteracy of a significant number of participants, verbal consent was obtained from the participants. The study protocol was reviewed and approved by the ethical committee of Shiraz University of Medical Sciences (SUMS No: 10908; date: March 2018).

#### The diabetes screening program (DSP) in Iran

**Fig. 1:**
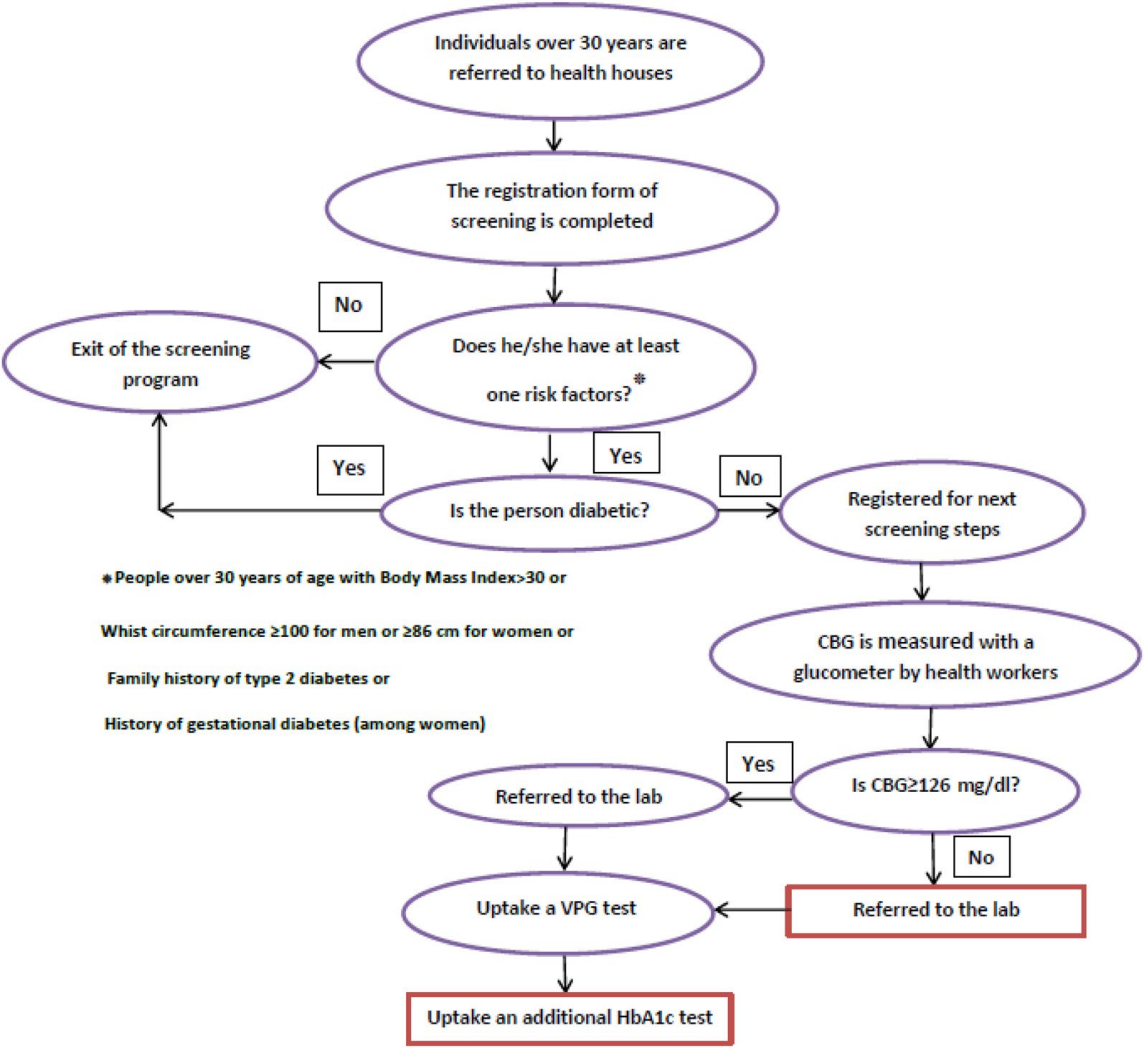
Flowchart of the Iranian diabetes screening program and the current study (rectangles represent additional steps taken by current study), *FBS* fasting blood glucose, *VPG* venous fasting plasma glucose, *HbA1c* glycosylated hemoglobin

Due to the high cost of treatment, the life-threatening complications, and relatively high prevalence of diabetes among the Iranian population, conducting effective screening programs to identify people with undiagnosed type 2 diabetes is of the utmost importance to the Iranian ministry of health. As a result, the Iranian ministry of health has recently implemented a universal and routine diabetes screening program into the national primary health care services. The program aims to detect undiagnosed diabetes cases among the high risk rural population who are older than 30 years of age. From 2016, DPS is being conducted by health centers and health houses to diagnose diabetes among those individuals over 30 years of age with at least one of the following risk factors: BMI ≥ 30, men with whist circumference ≥ 100 (or ≥ 86 cm for women), family history of type 2 diabetes and history of gestational diabetes (among women). In that regard, all rural residences aged over 30 years are to be annually screened for diabetes by the public health service providers (Fig. 1). Accordingly, the individuals are invited to the health houses to be visited by rural health nurses or voluntary health workers. The eligible individuals (those with one of the abovementioned risk factors) are asked to fast for at least 8 h prior to the morning that they have an appointment to visit the health house that they are registered with. In the health houses, the persons’ capillary fasting blood glucose (CBG) is measured with a glucometer. If the result of the CBG test is positive (CBG ≥ 126 mg/dl), the individual is referred to a health center to take a Venous fasting plasma glucose (VPG) test (as the diagnostic test). Also, HbA1c was conducted (as an additional step to DSP) considering %6.5 as a cut point for positive result. It is to be noted that HbA1c is not a part of routine procedure in the Iranian DSP. We added the test to the last phase of the screening procedure to define its validity in the Iranian population. A VPG test result (the gold standard) equal to or higher than 126 mg/dl is considered positive for type 2 diabetes [28].

Figure 1 presents the flowchart of the procedures of the Iranian DSP and the additional steps taken by the current study (shown in rectangles).

### Data collection

The present study recruited 1057 participants living in rural areas of Grash county. All participants were over 30 years of age and had at least one of the screening criteria defined by DSP (mentioned before). The participants were invited to the health houses and were interviewed by experienced and trained health nurses. The required data were collected via an interview-administered questionnaire that was specially designed for DSP and evaluated by the ministry of health. The questionnaire included demographic data and a history of type 2 diabetes among the participants or their relatives. In addition, CBG was measured with a glucometer (Easy Gluco, Infopia, Korea). The blood sample was taken from the tip of the middle finger of the left hand. After capillary fasting blood glucose test was conducted, irrespective of the result, the participants were referred to a public laboratory based in the nearby health center for having VPG test. The test was performed by an Alpha-Classic auto analyzer (Esfahan, Iran). In addition, at the same time, all participants had a HbA1C test that was conducted by a NycoCard Reader II (Alere/Axis-Shield, Oslo, Norway) device using the glucose oxidase method. Like many other studies that measured sensitivity and specificity of diabetes screening tests, this study followed WHO’s recommendations suggesting VPG as the gold standard for evaluation of capillary blood glucose and HbA1C tests [27].

Inclusion criteria: All participants were included providing they were over 30 years of age, had either of the above-mentioned risk factors, and reported no history of type 2 diabetes. Women were also to be not pregnant or breastfeeding.

Although, the dataset generated and/or analyzed during the current study is not publicly available due to being the intellectual property of Shiraz University of Medical Sciences, it is available from the corresponding author on reasonable request.

### Sampling and statistical methods

Sample size (*n* = 1010) was calculated based on the global prevalence of diabetes and using the formula provided by Karim Allah Hajian [29]. The formula is used to calculate the minimum required sample size for estimation of sensitivity and specificity of the diagnostic tests with a 5% marginal error. In practice, however, all individuals over 30 years of age with one or more of the previously mentioned risk factors were recruited (*n* = 1057).

The collected data were analyzed in SPSS 22 using frequency tables, cross-tabs, and chi-square tests. In addition, R4.0 was used to provide Receiver Operating Characteristic (ROC) curve to define the best cut points for CBS and HbA1c in screening type 2 diabetes among the study population.

## Results

In total, 1057 eligible individuals who were over 30 years of age and were living in rural areas of Gerash county participated in this study. The sex ratio (female/male) of the sample was 2 (*p* < 0.05), and almost similar age distributions were observed among the two genders (*p* > 0.05). The frequency distribution of the participants based on their test results is presented in Table 1. Accordingly, the rate of positive results of CBG (8.6%, setting ≥ 126 mg/dl as a cut point) and VPG (6.7%, setting ≥ 126 mg/dl as a cut point) were significantly different (*p* < 0.001). However, according to the results of HbA1c, the prevalence of type 2 diabetes was about 25% when the cut point was set at 6.5%, (as recommended by DSP) [28].

**Table 1:** Prevalence of type 2 diabetes based on the results of three diagnosis testes (CBG, VPG, and HbA1c)

Variable	*n* Total	*n*^a^ Suspected	%
CBG	1057	91	8.60
VPG	1057	71	6.70
HbA1c	1057	264	24.98

^a^Based on WHO recommended cut points (≥ 126 mg/dl for CBG and VPG and 6.50% for HbA1c)

### The validity of CBG and HbA1c tests

**Fig. 2:**
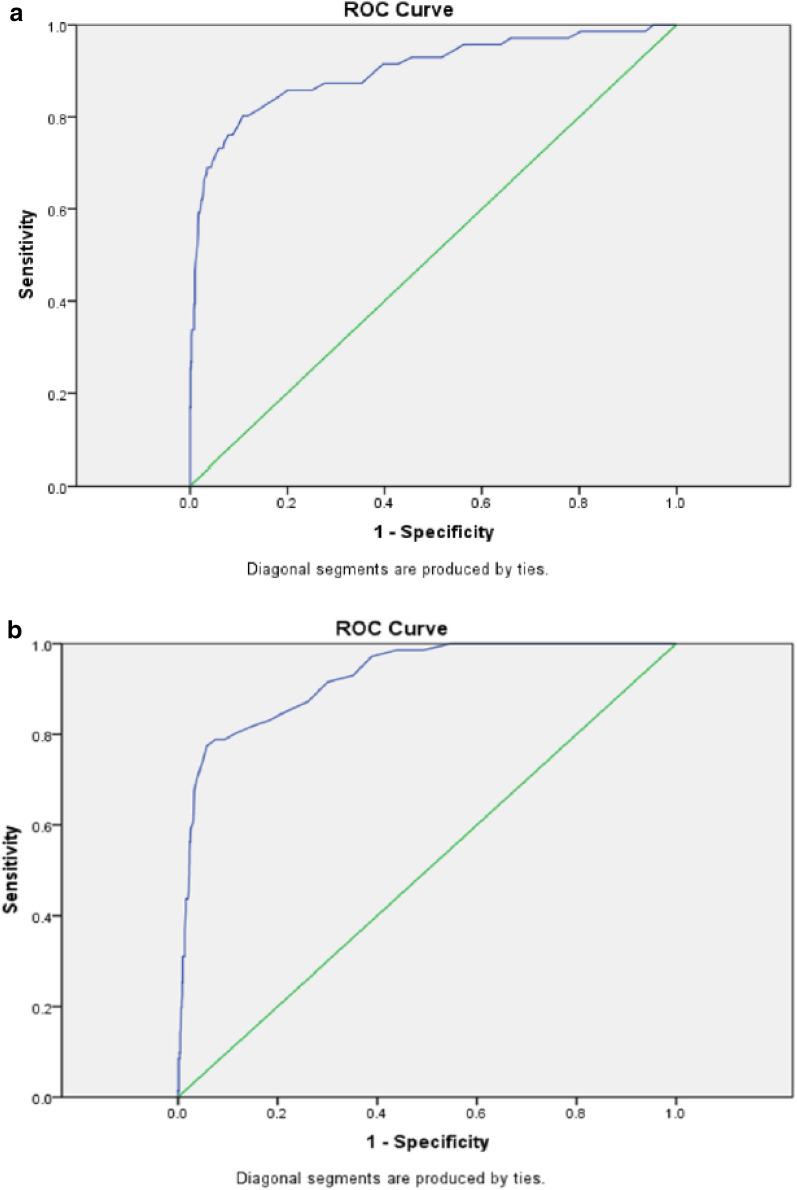
**a** ROC curve for CBG values as screening test; **b** ROC curve for HBA1c values as screening test; VPG was used as the gold standard; straight line represents the reference line

Using VPG (≥ 126 mg/dl) as the gold standard for diagnosis of type 2 diabetes, sensitivity, specificity, positive predictive values (PPV), and negative predictive values (NPV) for CBG and HbA1c are calculated and presented in Table 2. Additionally, as presented in Fig. 2, ROC analysis provided new cut points for the screening of diabetes based on CBG and HbA1c. Accordingly, at the DSP recommended cut points, the areas under the curve (AUC) were 88.6% and 92.8% for CBG and HbA1c, respectively. However, the estimated new cut points for the screening tests among the study population obtained by ROC analysis provided better performances (Table 3). Accordingly, the optimum cut points for CBG (116.50 mg/dl) and HbA1c (7.15%) are considerably different from those that are used by the Iranian DSP. Using these values as new cut points, sensitivity and specificity of CBG raised from 69.01 to 80.3% and decreased from 95.74 to 89.1%, respectively. Similarly, using 7.15% as the cut point for HbA1c, sensitivity and specificity changed from 84.5 to 77.5% and from 79.31 to 94.20%, respectively (Table 4).

**Table 2:** Sensitivity, specificity, positive predictive values (PPV), and negative predictive values (NPV) of CBG and HbA1c based on WHO’s recommended cut points

Screening variable	Clinical reference^a^	*n* Total	Sensitivity	Specificity	PPV	NPV
CBG	VPG^a^	1057	49/71 (69.01%)	944/986 (95.74%)	49/91 (53.84%)	944/966 (97.72%)
HbA1c^b^	VPG^a^	1057	60/71 (84.50%)	782/986 (79.31%)	60/264 (22.72%)	782/793 (98.61%)

*CBG* capillary fasting blood glucose, *VPG* venous fasting plasma glucose, *PPV* positive predictive value, *NPV* negative predictive value

^a^CBG ≥ 126 mg/dl; VPG ≥ 126 mg/dl and clinical diagnosis

^b^HbA1c ≥ 6.5%

**Table 3:** Receiver operating characteristic curve indexes for CBG and HbA1c vs. VPG

	CBG	HbA1c
Cut point (as recommended)	126 (mg/dl)	6.5 (%)
*n*. positive	91	264
*n*. negative	966	793
Area under the curve	0.902	0.925
SE	0.023	0.015
*p*-value	< 0.001	< 0.001
95% CI	0.856–0.948	0.896–0.954
Sensitivity	80.30	77.50
Specificity	89.10	94.20
Optimal cut point	116.50	7.15

*SE* standard error, *CI* confidence interval

**Table 4:** Sensitivity, specificity, PPV, and NPV for CBG and HbA1c vs. VPG based on cut points defined by ROC curve analysis

Screening test	Reference	*n*	Sensitivity	Specificity	PPV	NPV
CBG	VPG	1057	57/71 (80.28%)	879/986 (89.14%)	57/164 (34.75%)	879/893 (98.43%)
HbA1c	VPG	1057	55/71 (77.46%)	929/986 (94.21%)	55/112 (49.10%)	929/945 (98.30%)
*P*^a^			0.001	0.001		

CBG ≥ 116.50(mg/dl); VPG ≥ 126 (mg/dl) and clinical diagnosis

*PPV* positive predictive value, *NPV* negative predictive value

HbA1c ≥ 7.15; ^a^compared with indexes based on WHO’s recommendation cut points (CBG ≥ 126 mg/dl and HbA1c ≥ 6.5%)

## Discussion

Early detection of type 2 diabetes via an efficient screening program is essential in the prevention and management of the related life-threatening complications. Although several tests are introduced to diagnose diabetic patients, serious debates are still ongoing over the validity and reliability of their results [30–32]. It seems that the observed inconsistency in the validity of the test results is due to several reasons. For example, in a study conducted in India, a bimodal distribution of fasting CBG was observed. Obviously, this phenomenon could not be detected by CBG with a cut point between 140 and 120 mg/dl [33]. In addition, the American Diabetes Association has suggested that the glucose level of the patient’s blood changes over time depending on several factors including the disease progress [31]. In fact, it is possible that despite the presence of type 2 diabetes, the metabolic changes in the body are not big enough to detectably raise blood sugar [34]. CBG is a test that is more frequently used in type 2 diabetes screening programs due to its low cost and ease of use. However, in several studies, the validity of the results of CBG test has been questioned [32–35]. Technical issues as well as environmental, psychological, and medical conditions are listed as potential factors that affect the validity of the results of the test run by a glucometer [32].

Table 5 shows a comparison of the validity of different diabetes screening strategies in different countries. According to what was reported by Benja Muktabhant et al. [23], the sensitivity, specificity, and positive and negative predictive values for CBG (81.4%, 97.8%, 71.4%, and 98.7%, respectively) and HbA1c (39.70%, 96.70%, 56.80%, and 93.70%, respectively) were, to some extent, different from the corresponding indexes reported by the current study, when the VPG test result was used as the gold standard (Table 5). In another study, the researchers used HbA1c as a screening test and announced sensitivity, specificity, and positive and negative predictive values as 84.50%, 79.31%, 22.72%, and 98.61%, respectively [36]. However, Farahani et al. [37] compared HbA1c results with VPG as the gold standard test (with a cut point at 110 dl/ mg) and obtained 100% sensitivity, 12.50% specificity, 82.10% positive, and 66.70% negative predictive values, figures that are fundamentally different from those reported by the current study.

**Table 5:** Comparison of the validity of different diabetes screening strategies

Reference number	Country	Population screened	Screening test	Sensitivity (%)	Specificity (%)	Cut-point	AUC%
	Current study	People over 30 years of age with BMI > 30 whist circumference ≥ 100 for men or ≥ 86 cm for women, (*n* = 1057)	Capillary fasting blood glucose with glucometer	69.0	95.7	116.5	90.2
	Current study	The same as above	Glycated haemoglobin (HbA1c)	84.5	79.3	7.2%	92.5
[42]	India	People over 20 years of age (*n* = 2350)	Glycated haemoglobin (HbA1c)	93.3	92.3	6.4%	96.0
[43]	USA	People over 30 years of age (*n* = 5395)	Gglycated haemoglobin (HbA1c)	43.0	99.2	6.5%	86.0
[23]	Thailand	People over 35 years of age and older (*n* = 669)	Capillary fasting blood glucose with glucometer	45.6	96.3	91.0%	75.0
[23]	Thailand	People over 35 years of age and older (*n* = 669)	Capillary fasting blood glucose with glucometer	81.4	97.8	101	89.0
[44]	South Africa	People over 16 years of age and older (*n* = 946)	Fasting blood glucose	50.0	95.0	6.1	85.0
[45]	South Africa	People over 18 years of age (*n* = 1190)	Gglycated haemoglobin (HbA1c)	74.1	98.1	6.0	95.0%
[27]	Arab and European population (meta-analysis)		Gglycated haemoglobin (HbA1c)	42.0	97.0	6.5	84.0%

*BMI* body mass index

It is to be noted that sensitivity and specificity are independent of the prevalence of the disease in a population, but the positive predictive value increases when prevalence increases, and negative predictive value increases when the prevalence of the disease decreases. As a result, the predictive values of a test in a community are not comparable to those communities with different prevalence rates [38].

As recommended by the World Health Organization and the American and European Diabetes Associations, HbA1c is commonly used for screening or clinical diagnosis of diabetes [9, 39]. In Iran, following the World Health Organization, HbA1c test is used for the diagnosis of diabetes with a cut point at 6.50% [28]. However, several studies suggested significant contradictions in the results of the test. For example, according to a report from the International Expert Committee for Diagnosis and Classification of Diabetes, HbA1c test results may be affected by conditions such as hemoglobinopathies, pregnancy, uremia, blood transfusion, hemolytic anemia, and also by the applied laboratory methods [30]. In addition, according to a study, when compared to the two-hour blood glucose level and fasting blood glucose, the results of HbA1c were less accurate in identifying people who were at risk of diabetes [40]. Moreover, the use of HbA1c is costlier and requires sophisticated laboratory facilities that are hardly affordable by many developing countries [17]. Despite these, Nathan et al. [41] recommended HbA1c test results as a type 2 diabetes care assessment measure because they found a strong correlation between the test results and the diabetic complications.

The current evaluation study on 1057 participants with no history of diabetes, measured the validity of DSP in the Iranian population. Based on the results, when CBG (with a cut point at 126 mg/dl) as a screening test and VPG (with a cut point at 126 mg/dl) as a gold standard test were applied, sensitivity, specificity, and positive predictive value of the tests appeared to be low when compared to the corresponding results from other countries. However, when the performance of the Iranian DSP is compared with the results of studies from European (AUC = 0.844) and Arab (AUC = 0.847) countries, the performance of HbA1c and CBG for the Iranian DSP is significantly better (AUC = 0.925 for HbA1c and AUC = 0.902 for CBG) [27]. However, using the CBG test, 30.99% of people with type 2 diabetes were not detected, while 4.26% of the healthy subjects underwent unnecessary clinical and laboratory procedures (false positive). Based on the results of the current study, the recommended cut point for optimal sensitivity and specificity for CBG was calculated at 116.50 mg/dl. When HbA1c was used as a screening test, 15.50% of diabetes patients were not detected, while 20.69% of the healthy subjects underwent unnecessary clinical and laboratory procedures (false positive). In that regard, the best cut point for HbA1c was at 7.15%, with sensitivity and specificity of 77.50% and 94.20%, respectively.

Compared to several other countries, the performance of the Iranian DSP is relatively better (Table 5). This may be due to the differences in the defined criteria for selecting populations for screening (high-risk groups) in different countries. In the current study, ROC analysis suggested new cut points for even better performance of DSP in Iran. Our results may suggest the importance of locally defined cut points for type 2 diabetes tests as we need to take in to account the relevant differences between communities. Further studies are needed to understand different aspects of the suggested cut points and the risk factors selected by DSP to define the high-risk population to achieve a better performance of the program.

### Implications for policy and practice

This study evaluated the performance of diabetes screening of Iran using a population-based sampling method.

All procedures, instruments, and personnel used in this study were similar to those used by the national screening program making the results more representative and applicable.

New cut points are provided to increase the performance of the screening tests.

### Limitations

The participants in this study were all rural residents with lifestyles different to the urban population. As a result, our finding is better suited to be considered in urban populations.

## Data Availability

The datasets used and analyzed during the current study are available from the corresponding author on reasonable request.

## References

[1] Khan MAB, Hashim MJ, King JK, Govender RD, Mustafa H, Al KJ (2019). Epidemiology of type 2 diabetes—global burden of disease and forecasted trends. J Epidemiol Glob Health.

[2] Melmed S (2016). Williams textbook of endocrinology.

[3] Baghaei A, Sarrafzadegan N, Rabiei K, Gharipour M, Tavasoli AA, Shirani S (2010). How effective are strategies for non-communicable disease prevention and control in a high risk population in a developing country? Isfahan Healthy Heart Programme. Arch Med Sci.

[4] Faramarzi H, Bagheri P, Bahrampour A, Halimi L (2011). The comparison of prevalence of diabete and hypertension between rural areas of fars and rural area of EMRO region. Iran J Endocrinol Metab.

[5] Salem Z, Neshat A, Bagherian K, Sheykh Fath Elahi M, Sajadi M (2004). Prevalence of type II diabetes mellitus in over 30 year old population of Rafsanjan city in the year 2000. J Rafsanjan Univ Med Sci.

[6] Shah AD, Langenberg C, Rapsomaniki E, Denaxas S, Pujades-Rodriguez M, Gale CP (2015). Type 2 diabetes and incidence of cardiovascular diseases: a cohort study in 1.9 million people. Lancet Diabetes Endocrinol.

[7] Tancredi M, Rosengren A, Svensson A-M, Kosiborod M, Pivodic A, Gudbjörnsdottir S (2015). Excess mortality among persons with type 2 diabetes. N Engl J Med.

[8] Zhou B, Lu Y, Hajifathalian K, Bentham J, Di Cesare M, Danaei G (2016). Worldwide trends in diabetes since 1980: a pooled analysis of 751 population-based studies with 4.4 million participants. Lancet.

[9] Jia W (2016). Standardising HbA1c-based diabetes diagnosis: opportunities and challenges. Expert Rev Mol Diagn.

[10] Ghaem H, Daneshi N, Riahi S, Dianatinasab M (2018). The prevalence and risk factors for diabetic retinopathy in Shiraz, Southern Iran. Diabetes Metab J.

[11] Dabelea D, Mayer-Davis EJ, Saydah S, Imperatore G, Linder B, Divers J (2014). Prevalence of type 1 and type 2 diabetes among children and adolescents from 2001 to 2009. JAMA.

[12] International Diabetes Federation IDF Diabetes Atlas 2012. 6th edition. http://www.idf.org/diabetesatlas.

[13] Gupta SK, Singh Z, Purty AJ, Vishwanathan M (2009). Diabetes prevalence and its risk factors in urban Pondicherry. Int J Diabetes Dev Ctries.

[14] Gupta SK, Singh Z, Purty AJ, Kar M, Vedapriya D, Mahajan P (2010). Diabetes prevalence and its risk factors in rural area of Tamil Nadu. Indian J Community Med.

[15] Seuring T, Archangelidi O, Suhrcke M (2015). The economic costs of type 2 diabetes: a global systematic review. Pharmacoeconomics.

[16] Azizi T, Harati H, Mirbolooki M, Saadat N, Azizi F (2005). Association of different anthropometric measures and type 2 diabetes in an Iranian urban population. Iran J Endocrinol Metab.

[17] World Health Organization. Global report on diabetes. Geneva: World Health Organization. 2016. https://www.who.int/news-room/fact-sheets/detail/diabetes. Accessed 21 July 2017.

[18] Ekoe J-M, Goldenberg R, Katz P (2018). Screening for diabetes in adults. Can J Diabetes.

[19] Harris MI, Klein R, Welborn TA, Knuiman MW (1992). Onset of NIDDM occurs at least 4–7 yr before clinical diagnosis. Diabetes Care.

[20] Porta M, Curletto G, Cipullo D, de la Longrais RR, Trento M, Passera P (2014). Estimating the delay between onset and diagnosis of type 2 diabetes from the time course of retinopathy prevalence. Diabetes Care.

[21] Papatheodorou K, Banach M, Edmonds M, Papanas N, Papazoglou D (2015). Complications of diabetes. Hindawi.

[22] Mathur D, Santoyo-Olsson J, Stewart A, Freyre R, Grossman M, Saavedra J (2008). Using capillary blood glucose for eligibility screening in community-based diabetes prevention study. Diabetes.

[23] Muktabhant B, Sanchaisuriya P, Sarakarn P, Tawityanon W, Trakulwong M, Worawat S (2012). Use of glucometer and fasting blood glucose as screening tools for diabetes mellitus type 2 and glycated haemoglobin as clinical reference in rural community primary care settings of a middle income country. BMC Public Health.

[24] Bhavadharini B, Mahalakshmi MM, Maheswari K, Kalaiyarasi G, Anjana RM, Deepa M (2016). Use of capillary blood glucose for screening for gestational diabetes mellitus in resource-constrained settings. Acta Diabetol.

[25] WHO. Diabetes country profiles 2016. https://www.who.int/diabetes/country-profiles/irn_en.pdf?ua=1. Accessed 5 Aug 2019.

[26] Zakerkish M, Rahimi N, Marefati H, Kovsarian Z (2013). Evaluation of cardiovascular risk factors in diabetic patients in two regions with differences in race, culture and climate. Jundishapur Sci Med J.

[27] Bertran E, Berlie H, Taylor A, Divine G, Jaber L (2017). Diagnostic performance of HbA1c for diabetes in Arab vs. European populations: a systematic review and meta-analysis. Diabetic Med.

[28] Yavari P (2014). Diabetes. Epidemiology textbook of prevalence disease in Iran.

[29] Hajian-Tilaki K (2014). Sample size estimation in diagnostic test studies of biomedical informatics. J Biomed Inform.

[30] Genuth S, Alberti KG, Bennett P, Buse J, Defronzo R, Kahn R (2003). Follow-up report on the diagnosis of diabetes mellitus. Diabetes Care.

[31] Sacks DB (2011). A1C versus glucose testing: a comparison. Diabetes Care.

[32] Tonyushkina K, Nichols JH (2009). Glucose meters: a review of technical challenges to obtaining accurate results. J Diabetes Sci Technol.

[33] Rushforth NB, Miller M, Bennett PH (1979). Fasting and two-hour post-load glucose levels for the diagnosis of diabetes. The relationship between glucose levels and complications of diabetes in the Pima Indians. Diabetologia.

[34] American Diabetes Association (2014). Diagnosis and classification of diabetes mellitus. Diabetes Care.

[35] Parwaiz M, Lunt H, Florkowski CM, Logan FJ, Irons L, Perwick C (2014). Assessment of glucose meter performance at the antenatal diabetes clinic: exploration of patient-related and pre-analytical factors. Ann Clin Biochem.

[36] Lorenzo C, Wagenknecht LE, Hanley AJG, Rewers MJ, Karter AJ, Haffner SM (2010). A1C between 5.7 and 6.4% as a marker for identifying pre-diabetes, insulin sensitivity and secretion, and cardiovascular risk factors: the insulin resistance atherosclerosis study (IRAS). Diabetes Care.

[37] Farahani H, Naeimi A (2005). Comparison of glycosylated hemoglobin and oral glucose tolerance test in diagnosis of diabetes in person with impaired fasting glucose. Arak Med Univ J.

[38] Mausner J, Kramer S, Janghorbani M (2010). Screening for disease detection. An introductory text epidemiology.

[39] WHO (2011). Report of a World Health Organisation Consultation: use of glycated haemoglobin (HbA1c) in the diagnosis of diabetes mellitus. Diabetes Res Clin Pract.

[40] Sumner AE, Thoreson CK, O'Connor MY, Ricks M, Chung ST, Tulloch-Reid MK (2015). Detection of abnormal glucose tolerance in Africans is improved by combining A1C with fasting glucose: the Africans in America Study. Diabetes Care.

[41] Nathan DM, Buse JB, Davidson MB, Ferrannini E, Holman RR, Sherwin R (2009). Medical management of hyperglycaemia in type 2 diabetes mellitus: a consensus algorithm for the initiation and adjustment of therapy: a consensus statement from the American Diabetes Association and the European Association for the Study of Diabetes. Diabetologia.

[42] Mohan V, Vijayachandrika V, Gokulakrishnan K, Anjana RM, Ganesan A, Weber MB (2010). A1C cut points to define various glucose intolerance groups in Asian Indians. Diabetes Care.

[43] Guo F, Moellering DR, Garvey WT (2014). Use of HbA1c for diagnoses of diabetes and prediabetes: comparison with diagnoses based on fasting and 2-hr glucose values and effects of gender, race, and age. Metab Syndr Relat Disord.

[44] Zemlin AE, Matsha TE, Hassan MS, Erasmus RT (2011). HbA1c of 6.5% to diagnose diabetes mellitus—does it work for us? The Bellville South Africa study. PLoS ONE.

[45] Hird TR, Pirie FJ, Esterhuizen TM, O'Leary B, McCarthy MI, Young EH (2016). Burden of diabetes and first evidence for the utility of HbA1c for diagnosis and detection of diabetes in urban black South Africans: the Durban diabetes study. PLoS ONE.

